# Response of native (*Quercus robur* L.) and alien (*Quercus rubra* L.) species to water stress and nutrient input in European temperate ecosystems

**DOI:** 10.1111/ppl.70070

**Published:** 2025-01-20

**Authors:** Morena Rolando, Paola Ganugi, Francesca Secchi, Daniel Said‐Pullicino, Eleonora Bonifacio, Luisella Celi

**Affiliations:** ^1^ University of Turin Department of Agricultural, Forest and Food Science Grugliasco Italy

## Abstract

Drought and nutrient‐poor soils can increase the invasive potential of non‐native species, further changing the ecosystems they invade. The high adaptability of these alien species, especially in their efficient use of resources, improves their resilience against abiotic stress. Here, we evaluated the response of the North American *Quercus rubra* L. (RO) and the European *Quercus robur* L. (EO) oak species to drought and nutrient scarcity as single and combined factors. Both species were grown under well‐watered or alternating short dry‐wet phases, with or without the addition of phosphorous (P) and labelled nitrogen (N). Leaf gas exchanges and stem water potential were measured; moreover, leaf chemical characterization was carried out. Under concurrent low fertility and drought, both species reduced gas exchanges and stem water potential, although RO recovery was faster than EO. Nutrient inputs did not modulate RO's physiological response; however, P supply increased its uptake of the more available N forms (^15^NH_4_^15^NO_3_). The different leaf contents of N and P demonstrated that EO has lower nutrient use efficiency compared to RO. Nevertheless, P addition significantly mitigated the drought effects on EO, highlighting the crucial role of this nutrient in aiding EO's recovery under stress conditions. RO invasive potential may be linked to its superior adaptability and resource‐use efficiency under combined abiotic stress. Nevertheless, EO competitiveness can be improved through targeted nutrient management.

## 1 INTRODUCTION

Increasing ambient temperatures and alterations in water regimes due to climate change strongly threaten the equilibrium of many terrestrial ecosystems. In particular, the intensification of more prolonged and frequent drought events may alter the geographical distribution and growth of plants (Scheffers et al., 2016; Dyderski et al., 2018), favouring the invasion by alien species, which often outperform native ones in terms of water and nutrients use efficiencies. The competitiveness of these species versus the autochthonous vegetation may, therefore, be exacerbated by climate change, especially where and when soil nutrient resources are scarce.

Water deficit affects plant physiological processes related to nutrient and water uptake, photosynthesis, and assimilate partitioning, thereby impairing plant health and growth. To limit water loss, plants can close stomata, consequently decreasing leaf gas exchanges, such as stomatal conductance (gs), net photosynthesis (An) and leaf transpiration (E) (Farooq et al., 2012). However, the effect of drought on plants depends on the duration and intensity of the event; mild water stress reduces the gas exchanges, partially affecting photosynthesis. The increasing duration and magnitude of the event lead to complete stomatal closure, limiting CO_2_ assimilation and the further impairment of plant metabolisms. As a matter of fact, severe drought can cause oxidative stress and strongly decouple the processes related to photosynthesis (Zhou et al., 2007; Asrar & Elhindi, 2011). In turn, the decrease in CO_2_ assimilation, combined with the water deficit, may reduce nutrient plant uptake (Cramer et al., 2009; Waraich et al., 2011; Sardans & Peñuelas, 2012; He & Dijkstra, 2014). Plant nutrient uptake is also affected by their mobility and availability in soil. As regards nitrogen (N), its transport is primarily driven by mass flow (Hinsinger, 2001) and slightly impacted by drought (Lambers et al., 2008). Although soil N availability decreases as soil water content declines, mainly due to the reduced decomposition of N‐bearing organic compounds, the initial soil fertility has a marginal effect on N uptake during drought (Meisser et al., 2019). However, the appropriate N content in plant tissues may alleviate the negative effect of water deficit on plants, improving their water use efficiency and photosynthetic capacity (Reich et al., 2009; Querejeta et al., 2022). Additionally, N limits the photooxidation of chloroplast pigments, stimulating the production of antioxidants, soluble sugars and carotenoids (Song et al., 2019).

Phosphorus (P) mobility occurs through soil diffusion (Hinsinger, 2001). Therefore, the impact of drought on P mobility is much stronger than that on N (Lambers et al., 2008). In addition, soil P availability decreases as soil water content declines due to a larger extent of adsorption/precipitation processes on minerals, and this process is even enhanced across fertility gradients, further reducing plant P uptake (Mariotte et al., 2020). Therefore, in rich P soils, the lower P content in plants may be related to a direct effect of water deficit on plant uptake, while in less fertile soils, drought can further reduce plant uptake due to the strong retention by the mineral phase, exacerbated by the competition between plants and microbes for P (Meisser et al., 2019).

Nutrient addition may mitigate the combined stress determined by drought and nutrient scarcity. In low fertility soils, P input has been reported to modulate the stomatal conductance (Brück et al., 2000), cell‐membrane stability (Sawwan et al., 2000), An (Ackerson, 1985), and stem water potential (Ψ_stem_) (Radin & Eidenbock, 1984; Singh et al., 1997), improving the drought tolerance of plants. Additionally, appropriate P supply may promote the synthesis of osmolytes and antioxidant agents, which are important defences against dry conditions (Tariq et al., 2018). Generally, drought‐induced nutrient limitation does not occur whether the soil nutrient supply exceeds the plant demand. Conversely, drought indirectly affects plant growth when nutrient availability is lower than plant demand (Gonzalez‐Dugo et al., 2010; Meisser et al., 2019). Therefore, under dry conditions, plants are expected to be co‐limited by drought and nutrients in low‐fertility soils, while in nutrient‐rich soils, plants are primarily limited by water scarcity (Hooper & Johnson, 1999).

In European forests, one of the most widespread alien trees is *Quercus rubra* L. (Red Oak, RO), a Northern American species introduced at the end of the 17th century as an ornamental and commercially important tree used for timber production (Lambdon et al., 2008). Since its introduction, it has been recorded in 21 European countries, spanning from Portugal and Ireland in the West to Ukraine and European Russia in the East, and from Norway and Sweden in the North to Spain, Greece, and Italy in the South (GBIF Secretariat, 2022; Nicolescu et al., 2020, Lambdon et al. 2008). In Italy, the Po River plain (North Italy) was used to be covered with the *Querco‐carpinetum* coenosis, a forest composed mainly of *Carpinus betulus* L. and *Quercus robur* L. (English Oak, EO). In the last decades, the native coenosis has been replaced by RO due to its rapid growth and thick canopy, limiting the establishment of seedlings from other oak species and hampering the survival of the remaining nuclei of the original forest. The invasive potential of RO depends on site conditions and is limited in fertile soils but increases in dry and nutrient‐poor soils (Bonifacio et al., 2015; Lavnyy & Savchyn, 2016; Hasenauer et al., 2017). The water and nutrient demand of EO is higher than that of RO, therefore drought may compromise the competitiveness of the native species against the alien plant because of the direct effect of water deficit on plants and the indirect effect of water scarcity on nutrient availability (Timbal & Dreyer, 1994; Dyderski et al., 2018). However, the response of EO and RO to combined stresses, such as drought and nutrient scarcity, has been scarcely investigated.

The aim of our study was to assess the combined effects of drought and nutrient scarcity in EO forests invaded by RO. To reach this aim, a mesocosm experiment was set up by growing saplings of EO and RO in soils that were poor in nutrients and collected in an area fully colonized by RO. We hypothesize that (1) soil nutrient scarcity exacerbates drought effects, making RO more tolerant and better able to recover from short but intense dry events than EO due to its lower water and nutrient demands; (2) N and P inputs prevent drought‐induced nutrient limitations and mitigate the negative effect of water deficit in both species, particularly in EO; (3) under well‐watered conditions, EO has greater nutrient uptake compared to RO because of their different nutrient use efficiencies. To test these hypotheses, both EO and RO species received N and P fertilization, either alone or in combination, to manipulate the soil nutrient availability, whereas water stress was imposed through different water regimes. Nitrogen was added as ^15^NH_4_^15^NO_3_ to assess the different capacities of the plants to acquire N from added or native N soil sources.

## 2 MATERIALS AND METHODS

### 2.1 Plant and soil material

The experimental trial was carried out in a greenhouse located at the Department of Agricultural, Forest and Food Science (DISAFA) of the University of Turin, Italy. In December 2021, two‐year‐old *Quercus rubra* L. and *Quercus robur* L. plants, provided by a nursery, were transplanted into 3.4 L cylindrical pots (28 cm height × 12.5 cm diameter). A total of 80 plants (40 RO and 40 EO) were used in this study.

The soil (0–20 cm layer) was collected from La Mandria Natural Park, Northwestern Italy (N 45.153213, E 7.581204), within a pure RO forest, once covered by *Carpinus betulus* L. and EO. The soil, classified as Oxyaquic Fragiudalf (USDA, Soil Taxonomy), was very poor in available nutrients, as it can be deduced by its initial characteristics: pH 4.4, 29.3 g kg^−1^ of total organic C, 2.2 g kg^−1^ of total N, 5.2 mg kg^−1^ of available P (P_ols_), 4.16 mg N‐NO_3_^−^ kg^−1^ and 36 mg N‐NH_4_^+^ kg^−1^. In Bonifacio et al. (2015), more details about pedoclimatic features of the area are reported. Plants were grown under partially controlled conditions with average air temperature and relative humidity of 27.4 ± 5.5°C and 56.6 ± 16.3%, respectively. Plants were 59.6 ± 17.3 cm tall at the onset of the experiment.

### 2.2 Experimental design and treatments

The experimental design consisted of a split plot with two plant species (SP: EO and RO) as main plots, two water regimes (WM) as subplots, four nutrient inputs (NI) as sub‐subplots and five replicates per treatment (R). The two water regimes involved (1) a well‐water treatment (WW) in which 20 EO and 20 RO plants were regularly irrigated over the duration of the entire experiment (from 16 June to 3 August 2022) in order to keep the soil‐to‐pot capacity; and (2) a drought and recovery treatment in which 20 plants per species were exposed to three successive cycles of drought (D; water stress), lasting 7 days, followed by recovery (W; stress relief). The number of cycles was chosen according to He and Dijkstra (2014) in order to evaluate when and how species changed their response to dry conditions during consecutive drought events. Water deficit during the D phase was induced by stopping the addition of water, exposing plants to a short but intense drought stress. After each drought phase, the pots were re‐watered to re‐establish the soil water content similar to WW plants and maintained well‐watered for the subsequent 13 days before inducing the next cycle.

Nutrient supply involved the addition of N alone (+N‐P), P alone (‐N + P), N and P together (+N + P), and a non‐fertilizer original nutrient‐limited soil (‐N‐P) to serve as a control. In detail, on the 20th of April 2022, the mesocosms received the following nutrient input: 89.3 mg N kg^−1^ as isotopically enriched ^15^NH_4_^15^NO_3_ (10 at% ^15^N; +N‐P and + N + P treatments) and/or 39.3 mg P kg ^−1^ as KH_2_PO_4_ (‐N + P and + N + P treatments). All mesocosms also received 94.6 mg K kg^−1^ as KCl. The nutrient input was established based on the literature (Sabaté et al., 1992). Additionally, preliminary P adsorption curves in the soil were carried out to take into account the P fraction retained by soil minerals and ensure a sufficient available P pool.

### 2.3 Measurement of leaf gas exchanges and stem water potential

Leaf gas exchanges, including gs, An and E, were measured on fully expanded leaves exposed to direct sunlight using a portable infrared gas analyzer (ADC‐LCPro+ system, The Analytical Development Company Ltd.). CO_2_ values were maintained at greenhouse conditions (400–450 ppm). Leaf gas exchange was monitored every two days (between 10:00 am and 12:30 pm) on three to five plants in each treatment (one leaf per plant) for the whole duration of the experimental trial. The number of samples for the measurements was selected to allow us to work within this specific time window, minimizing variability in environmental conditions. Here, we reported data collected on the last day of each D treatment (DOY 174, 193 and 213) and the second day after recovery (DOY 176, 195 and 215). Meanwhile, leaves were collected at the end of each D phase for Ψ_stem_ measurements (DOY 174, 193 and 213). Leaves were placed in humidified aluminium foil‐wrapped plastic bags for about 15 min prior to excision. After excision, leaves were allowed to equilibrate for an additional 10 min before the water potential was measured using a Scholander‐type pressure chamber (Soil Moisture Equipment Corp.).

### 2.4 Chemical characterization of leaves

On the last day of each D treatment (DOY 174, 193 and 213), leaves were collected to analyze the total C and N, δ^15^N, P, potassium (K), calcium (Ca), magnesium (Mg), iron (Fe) and manganese (Mn). Total C, N and δ^15^N were determined by high‐temperature combustion and continuous‐flow isotope ratio mass spectrometry (Vario Isotope Select and Isoprime 100; Elementar Analysensysteme GmbH, Hanau, Germany). The instrument was calibrated relative to the atmospheric nitrogen (N_2_ Air) using standard reference materials IAEA‐600, IAEA‐603 and IAEA‐N2. The uncertainty of measurements was monitored by repeated measurements of internal laboratory standards and standard reference materials. Precision was determined to be 0.1‰. ^15^N enrichment in the applied fertilizer (atom%^15^N_*fertilizer*_) and leaves of labelled (+N‐P and + N + P) and non‐labelled (‐N‐P, ‐N + P) treatments were used to calculate the fraction of plant N derived from mineral inputs (NdI) according to the following equation:

(1) $$\mathrm{NdI}=\left(\frac{\;\text{atom}{\%}^{15}N_{\text{labeled}}-\text{atom}{\%}^{15}N_{\text{unlabeled}}}{\text{atom}{\%}^{15}N_{\text{fertilizer}}-\text{atom}{\%}^{15}N_{\text{unlabeled}}}\right)*100$$

Total K, Ca, Mg, Fe and Mn were determined by acid digestion with concentrated H_2_SO_4_ and HClO_4_ followed by inductively coupled plasma–optical emission spectroscopy (ICP‐OES, PerkinElmer Optima 5500). Total P was also determined on the same extracts spectrophotometrically by using the malachite green method (Ohno & Zibilske, 1991).

### 2.5 Data collection and statistical analyses

Data for stress and recovery were analyzed separately to better evaluate differences in the effects of different NI on EO and RO species subjected to water stress due to multiple interruptions and recoveries of water supply. All the statistical analyses were carried out using R 4.3.1 (R Code Team, 2022).

First, a four‐way analysis of variance (ANOVA) was performed with a mixed effect model on each physiological parameter and each leaf chemical element using the *nlme* package (Pinheiro et al., 2007). SP, WM, NI and T were selected as fixed factors, while R – represented by 3 out of 5 replicates – was the random effect of the mixed model. The distribution of measured variables was checked for normality and homogeneity of variances using the Shapiro–Wilk test and Levene's test, respectively. Data were log‐transformed prior to analysis when the ANOVA assumptions were violated; then, Shapiro–Wilk and Levene's tests were performed again on log‐transformed data. SP, WM, NI and T means were compared using Tukey's honestly significant difference (HSD) at *p‐*value <0.05 with *multcomp* packages (Hothorn et al., 2008).

The correlation between all the collected variables – physiological and chemical data – was assessed using a Spearman correlation model implemented in the *corrplot* package at a significance level *p‐*value ≤0.05.

Afterwards, a multivariate analysis of variance (MANOVA) for each water stress and recovery stage was conducted to determine ‐within each species‐ the influence of WM and NI on all physiological data simultaneously. The same analysis was carried out on chemical data.

Finally, the effective recovery capacity of EO and RO plants was assessed by the delta recovery rate (∆RR) of each physiological parameter (X) for each recovery stage (DOY_y_), calculated as follows:

(2) $$∆\mathrm{RR}\;X_{{\mathrm{DOY}}_{y}}=\left(∆X_{D⟶W\;{\mathrm{DOY}}_{y}}-{\bar{∆X\;}}_{\mathrm{WW}\;{\mathrm{DOY}}_{y}}\right)*100$$

where $∆X_{D⟶W\;{\mathrm{DOY}}_{y}}$and ${\bar{∆X\;}}_{\mathrm{WW}\;{\mathrm{DOY}}_{y}}$ represented the recoveries of X parameter on DOY_y_ of W and WW samples, respectively obtained with the following two formula:

(3) $$∆X_{D⟶W\;{\mathrm{DOY}}_{y}}=\frac{\left(X_{W\;{\mathrm{DOY}}_{y}}-X_{D\;{\mathrm{DOY}}_{z}}\right)}{X_{W\;{\mathrm{DOY}}_{y}}}$$

(4) $${\bar{∆X}}_{\mathrm{WW}\;{\mathrm{DOY}}_{y}}=\frac{\left(\begin{array}{c} \frac{\left(X_{\mathrm{WW}\;R1\;{\mathrm{DOY}}_{y}}-X_{\mathrm{WW}\;R1\;{\mathrm{DOY}}_{z}}\right)}{X_{\mathrm{WW}\;R1\;{\mathrm{DOY}}_{y}}}+\frac{\left(X_{\mathrm{WW}\;R2\;{\mathrm{DOY}}_{y}}-X_{\mathrm{WW}\;R2\;{\mathrm{DOY}}_{z}}\right)}{X_{\mathrm{WW}\;R2\;{\mathrm{DOY}}_{y}}}\\ +\frac{\left(X_{\mathrm{WW}\;R3\;{\mathrm{DOY}}_{y}}-X_{\mathrm{WW}\;R3\;{\mathrm{DOY}}_{z}}\right)}{X_{\mathrm{WW}\;R3\;{\mathrm{DOY}}_{y}}} \end{array}\right)}{3}$$

where $X_{W\;{\mathrm{DOY}}_{y}}$ was the X parameter of W sample on DOY_y;_

$X_{D\;{\mathrm{DOY}}_{z}}$ was the X parameter of D sample on the water stress stage (DOY_z_) prior to DOY_y_;

$X_{\mathrm{WW}\;R1\;{\mathrm{DOY}}_{y}}$, $X_{\mathrm{WW}\;R2\;{\mathrm{DOY}}_{y}}$ and $X_{\mathrm{WW}\;R3\;{\mathrm{DOY}}_{y}}$ were the X parameters of replicates 1, 2 and 3 of WW samples on DOY_y;_

$X_{\mathrm{WW}\;R1\;{\mathrm{DOY}}_{z}}$, $X_{\mathrm{WW}\;R2\;{\mathrm{DOY}}_{z}}$ and $X_{\mathrm{WW}\;R3\;{\mathrm{DOY}}_{z}}$ were the X parameters of replicates 1, 2 and 3 of WW samples on DOY_z._

## 3 RESULTS

### 3.1 Physiological parameters

#### 3.1.1 Stress treatments (DOY 174, 193 and 213)

**FIGURE 1:**
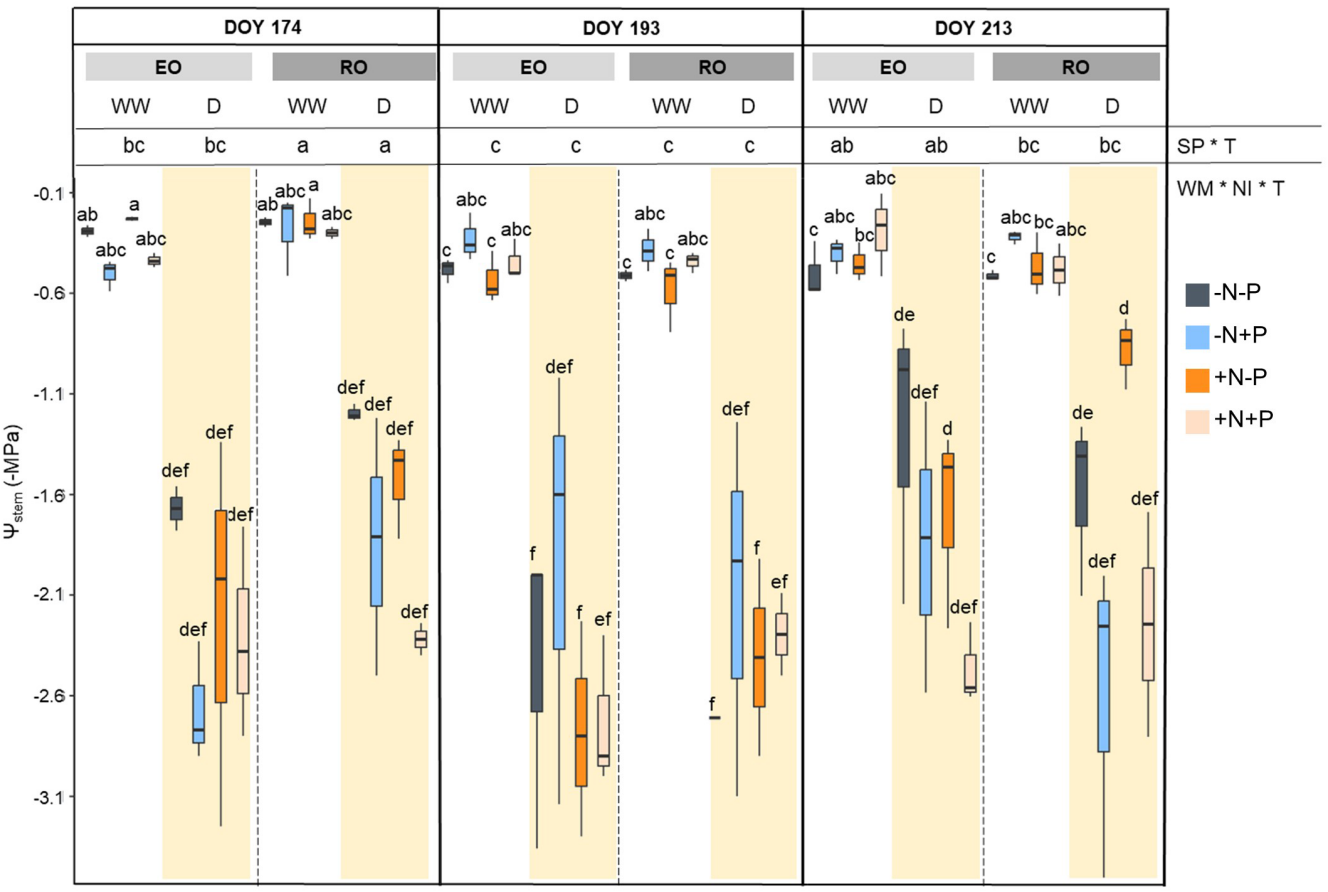
Stem water potential (Ψstem) of both species (SP; English Oak, EO and Red Oak, RO) under different water management (WM; well‐watered, WW and drought conditions, D) with different nutrient input (NI), during time (T). According with ANOVA analysis and Tukey's post ‐ hoc, the different letters denote significant differences among factors (*p* < 0.05).

Under well‐watered conditions, Ψ_stem_ values were similar for both species growing in nutrient‐poor soils, approximately −0.29 ± 0.03 MPa for EO WW and − 0.25 ± 0.02 MPa for RO WW (DOY 174, Figure 1). Over time, on DOY 193 and 213, Ψ_stem_ reached lower values. This drop is likely due to the measurements being conducted in summer, during a period of high atmospheric evaporative water demand, characterized by average daily temperatures >28°C and RH > 55%. However, the different nutrient inputs did not affect the stem water potential in well‐irrigated plants for either species. Under water stress, both species experienced a notable decrease in Ψ_stem_ (Figure 1). On DOY 174 (the seventh day of the first water stress), the Ψ_stem_ of nutrient‐stressed plants dropped to −1.67 ± 0.11 MPa for EO D and to −1.20 ± 0.04 MPa for RO D. Six days after the second water stress treatments (DOY 193), the interspecific differences in Ψ_stem_ of D plants disappeared because of a decrease in the stem water potential of RO. On DOY 213, the Ψ_stem_ of D plants was higher than previously (−1.82 MPa on average for both species).

**FIGURE 2:**
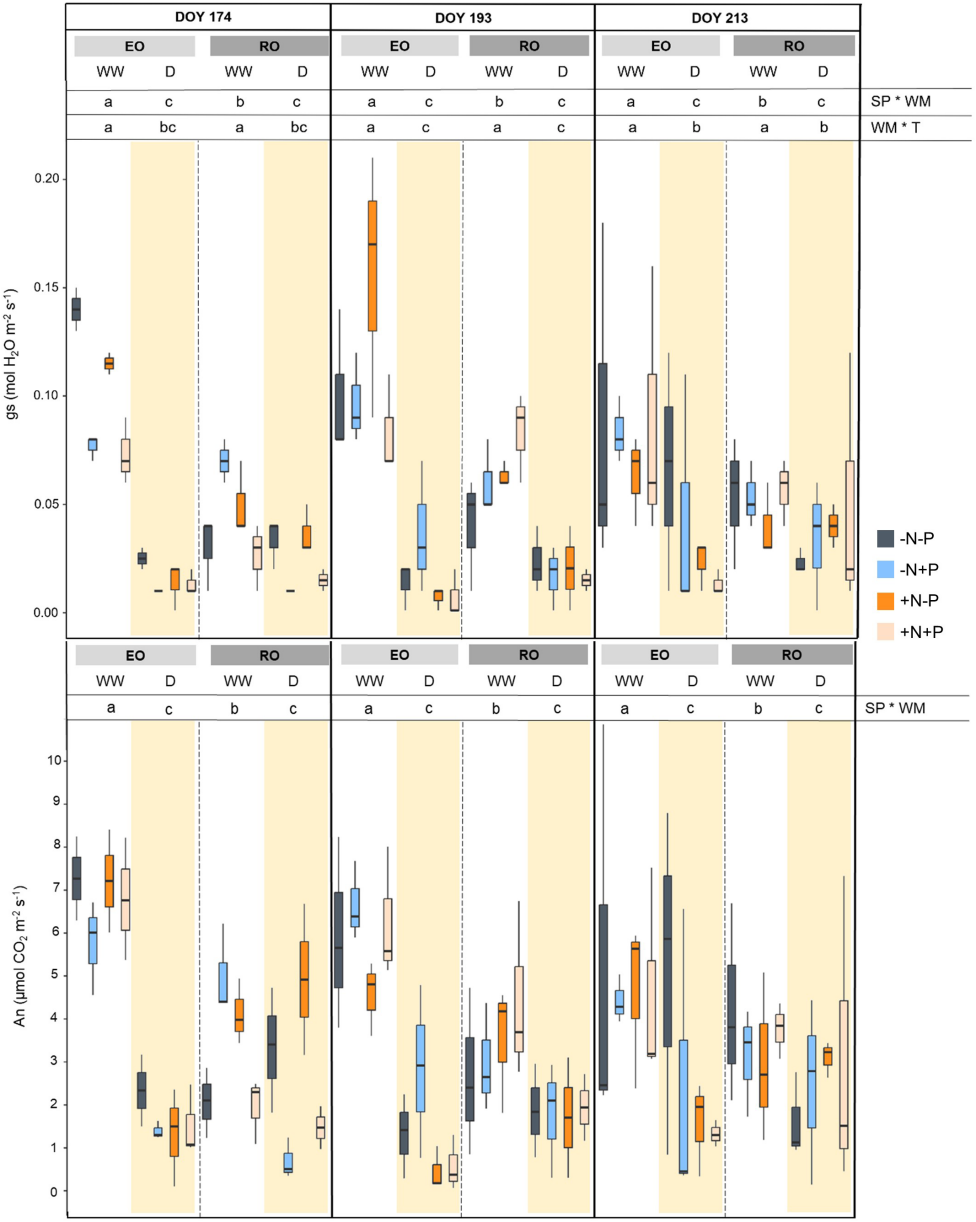
Stomatal conductance (gs) and CO2 leaf assimilation (An) of both species (SP; English Oak, EO and Red Oak, RO) under different water management (WM; well‐watered, WW and drought conditions, D) with different nutrient input (NI), during time (T). According with ANOVA analysis and Tukey's post ‐ hoc, the different letters denote significant differences among factors (*p* < 0.05).

The NI did not affect the Ψ_stem_ and leaf gas exchanges of both species during drought. The stomatal conductance and photosynthesis of EO WW were higher than those detected from RO WW for the whole duration of the experimental trial, thus indicating differences between species (Figure 2). Similar results were also obtained for E (Figure S1). Conversely, similar values of gs, E and An, for both EO D and RO D, were measured in each stress treatment. As a consequence, the reduction of gs, An, and E in EO from the well‐watered conditions to the stress ones were much more marked than in RO. Moreover, both drought‐stressed species exhibited higher leaf gas exchange values in DOY 213 than in DOY 193. In fact, ANOVA analysis showed a significant effect of WM*T interaction on gs and E (*p* < 0.05).

Afterwards, two‐way MANOVA was performed within the species and in each stress stage to simultaneously test the significance of WM and NI on all the physiological parameters measured (Table 1). The results revealed that, on DOY 174, WM and NI, as well as the interaction between these two factors, significantly affected the physiological parameters in both species. On DOY 193 and 213, all the physiological variables were affected only by WM, whereas a significant effect of WM*NI interaction (*p* < 0.05) was pointed out on DOY 213 in EO samples.

**TABLE 1:** Multivariate analysis (MANOVA) based on Pillai's Trace test of water management and nutrient input on physiological parameters (gs, E, An, Ψstem) of EO and RO species under different stress stages (DOYs 174, 193, 213).

Physiological parameters – stress								
Species	Time	Factor	Df	Pillai	approx F	num df	den df	P
EO	DOY 174	Water management (WM)	1	0.986	223.433	4	13	**7.703e‐12**
		Nutrient input (NI)	3	1.263	2.727	12	45	**0.007**
		WM * NI	3	1.153	2.341	12	45	**0.019**
	DOY 193	Water management (WM)	1	0.924	39.747	4	13	**3.594e‐07**
		Nutrient input (NI)	3	0.812	1.391	12	45	0.205
		WM * NI	3	0.493	0.737	12	45	0.708
	DOY 213	Water management (WM)	1	0.931	44.132	4	13	**1.924e‐07**
		Nutrient input (NI)	3	0.567	0.874	12	45	0.577
		WM * NI	3	1.043	1.998	12	45	**0.047**
RO	DOY 174	Water management (WM)	1	0.923	39.155	4	13	**3.929e‐07**
		Nutrient input (NI)	3	1.496	3.732	12	45	**6.13e‐4**
		WM * NI	3	1.225	2.587	12	45	**0.010**
	DOY 193	Water management (WM)	1	0.957	72.356	4	13	**9.445e‐09**
		Nutrient input (NI)	3	0.611	0.960	12	45	0.499
		WM * NI	3	0.608	0.954	12	45	0.504
	DOY 213	Water management (WM)	1	0.924	39.431	4	13	**3.768e‐07**
		Nutrient input (NI)	3	0.536	0.815	12	45	0.63357
		WM * NI	3	0.942	1.718	12	45	0.09471

#### 3.1.2 Recovery treatments (DOY 176, 195 and 215)

**FIGURE 3:**
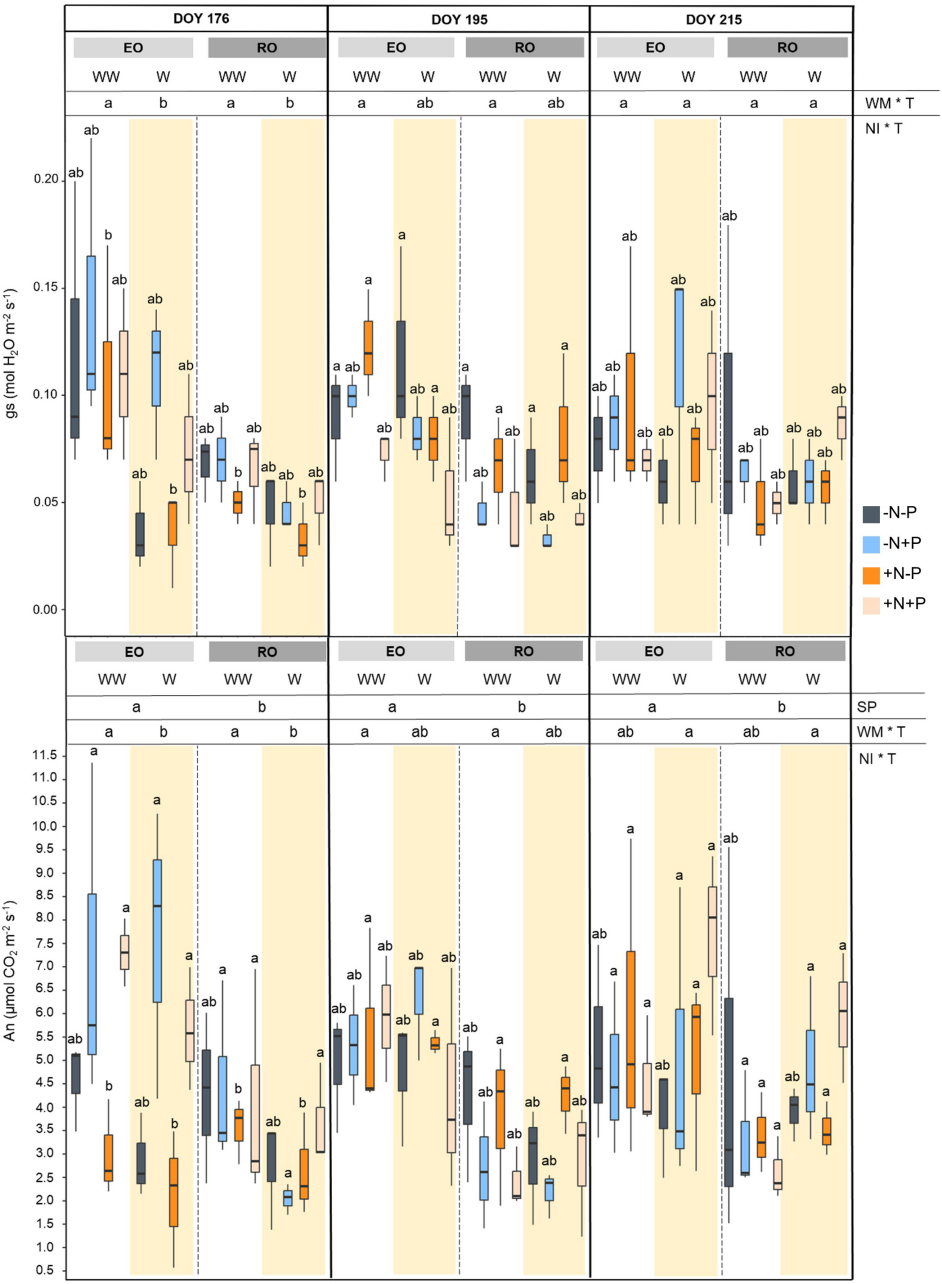
Stomatal conductance (gs) and CO2 leaf assimilation (An) of both species (SP; English oak, EO and Red oak, RO) under different water management (WM; well‐watered, WW and rewatered after drought, W) with different nutrient input (NI), during time (T). According with ANOVA analysis and Tukey's post ‐ hoc, the different letters show significant among factor (*p* < 0.05).

The ability of the two species to recover leaf gas exchanges after stress relief (recovery) and the timing of recovery differed between EO and RO. After two days of recovery from the first stress treatment (DOY 176), EO did not fully recover gs and An, while the values for RO were similar to those of well‐watered plants (Figure 3). On DOY 176, EO W plants under ‐N‐P and W + N‐P conditions displayed the lowest values of gs and An, which were comparable to the values measured at the end of D treatment (DOY 174). This suggests that N input did not promote the recovery process for EO plants. However, higher and similar values of gs and An were detected between EO W ‐N + P and EO WW ‐N + P, revealing a positive effect of P during the recovery (Figure 3). However, the N limited the P impact; in fact, on DOY 176, EO W + N + P showed positive percentage increments of leaf gas exchanges (gs: 82%; An: 73%; E: 66%) without reaching the parameters of well‐watered EO + N + P. NI did not affect the recovery of RO, and the gas exchanges were comparable to those of all nutrient input treatments.

After two days of water relief (recovery) from the second and third stress treatments (DOY 195, 215), plants completely restored their An and gs, reaching values similar to those measured in well‐watered EO and RO. The SP factor only showed significance on variable An (*p* < 0.05), highlighting the highest values in the native species.

The MANOVA analysis performed for each species demonstrated that the WM and NI factors did not have a significant effect on the global changes of leaf gas exchanges, nor did their interaction (Table 2). The only exception was observed on DOY 176, for which leaf gas exchanges of EO were influenced by the WM and NI factors at a high level of significance. The recovery capacity, i.e. ∆gs, and ∆E, showed a significant effect of NI*T interactions and allowed us to better follow the effects of nutrient inputs (Figure S3 & Figure S4). On DOY 176, EO W ‐N + P (44.2 ± 3.8%) and RO W ‐N + P (77.8 ± 2.8%) showed the highest ∆gs in both species (Figure S3). Conversely, during the second recovery time, the lowest ∆gs was detected in EO ‐N + P (26.4 ± 16.6%) and RO ‐N‐P treatment (17.6 ± 29.9%). Between DOY 195 and 215, a decrease in ∆gs was observed in both species. The ∆gs of EO was positive but below 50% in ‐N‐P (36.7 ± 6.1%) and + N + P (42.9 ± 22.3%), while negative values were observed in treatments where both N and P were added individually. During the last recovery period, there was no significant impact of NI on ∆gs of RO, and values below 25% were observed in each NI treatment.

**TABLE 2:** Multivariate analysis (MANOVA) based on Pillai's Trace test of water management and nutrient input on physiological parameters (gs, E, An) of EO and RO species under different recovery stages (DOYs 176, 195, 215).

Physiological parameters – recovery								
Species	Time	Factor	Df	Pillai	approx F	num df	den df	P
EO	DOY 176	Water management (WM)	1	0.471	4.149	3	14	**0.027**
		Nutrient input (NI)	3	1.087	3.029	9	48	**0.006**
		WM * NI	3	0.253	0.491	9	48	0.873
	DOY 195	Water management (WM)	1	0.148	0.812	3	14	0.508
		Nutrient input (NI)	3	0.624	1.401	9	48	0.214
		WM * NI	3	0.443	0.925	9	48	0.512
	DOY 215	Water management (WM)	1	0.041	0.199	3	14	0.895
		Nutrient input (NI)	3	0.760	1.809	9	48	0.090
		WM * NI	3	0.327	0.653	9	48	0.746
RO	DOY 176	Water management (WM)	1	0.301	2.012	3	14	0.159
		Nutrient input (NI)	3	0.371	0.754	9	48	0.658
		WM * NI	3	0.369	0.748	9	48	0.663
	DOY 195	Water management (WM)	1	0.046	0.227	3	14	0.876
		Nutrient input (NI)	3	0.582	1.284	9	48	0.270
		WM * NI	3	0.167	0.316	9	48	0.966
	DOY 215	Water management (WM)	1	0.288	1.890	3	14	0.177
		Nutrient input (NI)	3	0.150	0.282	9	48	0.976
		WM * NI	3	0.480	1.017	9	48	0.440

Concerning ∆E, the EO value on DOY 176 was higher in +N‐P (105.3 ± 64.7%) than in other NI treatments, which had values below 60%. Conversely, the ∆E of RO ‐N + P was 98.6 ± 6.5%, while ‐N‐P and + N‐P were respectively −72.2 ± 61.7% and − 32.9 ± 83.8% (Figure S4). Consistent with ∆gs, in the second recovery period, the smallest ∆E of EO was observed in ‐N + P (34.8 ± 17.0%), while the values of the other NI treatments exceeded 60%, reaching 180.8 ± 4.5% (in +N‐P). Conversely, at the same time, the lowest ∆E of RO was found in ‐N‐P (13.7 ± 18.8%), while +N‐P (79.8 ± 27.7%) and + N + P (88.9 ± 20.2%) exhibited the highest values within the species. On DOY 215, both species show smaller ∆E values in ‐N + P (EO = −42.6 ± 79.8%; RO = −5.7 ± 16.3%) and + N‐P (EO = −18.5 ± 30.8%; RO = 3.4 ± 20.8%) compared to ‐N‐P (EO = 26.3 ± 10.0%; RO = 21.9 ± 15.6%) and + N + P (EO = 33.1 ± 30.6%; RO = 26.8 ± 3.8%).

The ∆An of EO was higher than RO during all three recovery treatments, regardless of WM and NI. As a matter of fact, univariate ANOVA identified significant differences for SP (Figure S5). On DOY 176, ∆An of EO + N + P tended to be lower than the other NI treatments, even though no significant differences was detected for NI. During the same time period, RO exhibited a high ∆An in ‐N + P (80.6 ± 10.1%). Between DOY 176 and 195, +N‐P inclined to increase ∆An in both species, while in EO, only ‐N + P reduced ∆An. Conversely, on DOY 195, ∆An in RO tended towards 0% in all NI treatments except +N‐P (41.6 ± 25.6%). Despite NI, the ∆An of EO disposed to be lower on DOY 215 than on DOY 195, as well as RO + N‐P.

### 3.2 Leaves chemical characteristics

**FIGURE 4:**
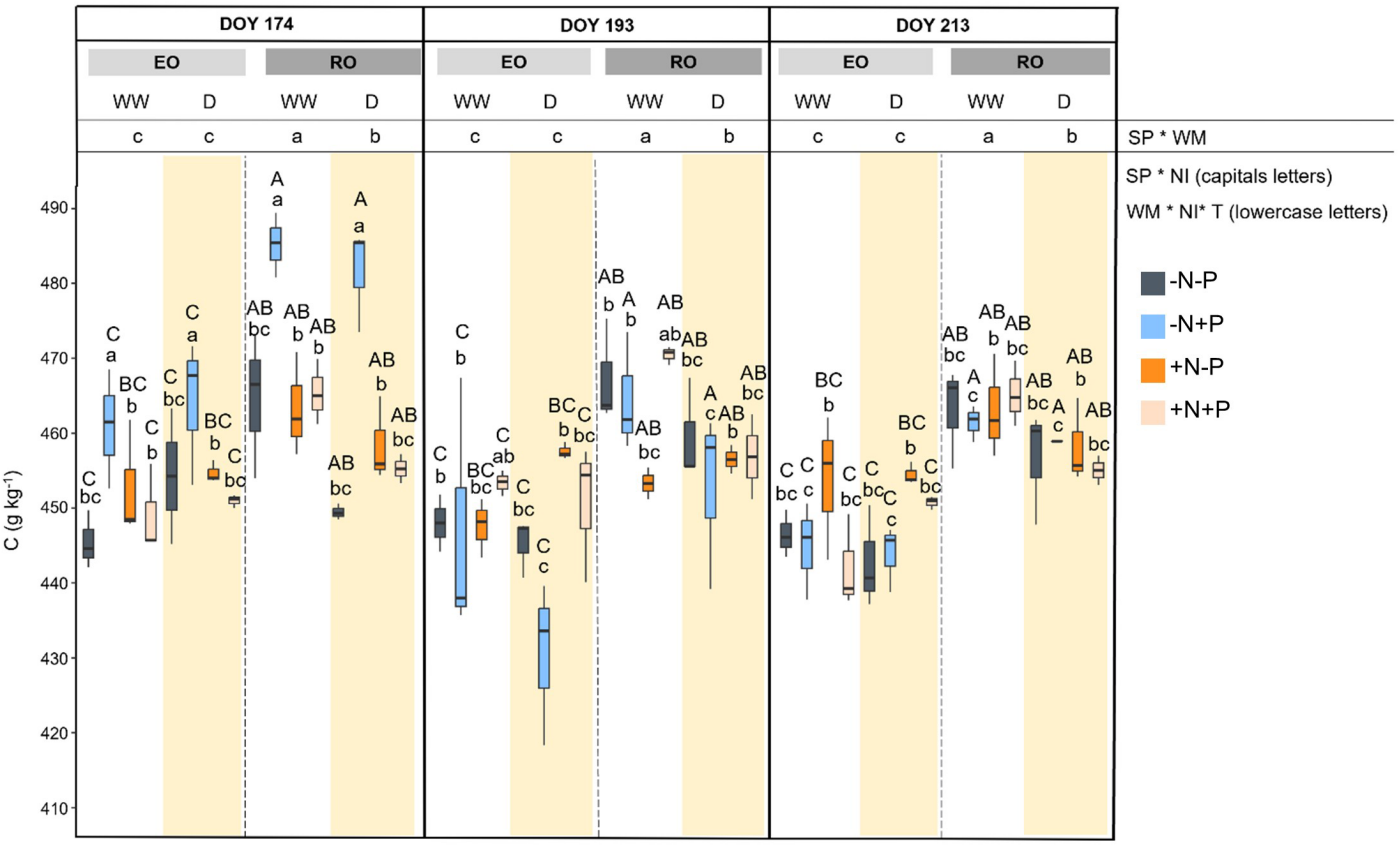
Carbon content (C) in leaves of both species (SP; English Oak, EO and Red Oak, RO) under different water management (WM; well‐watered, WW and drought conditions, D) with different nutrient input (NI), during time (T). According with ANOVA analysis and Tukey's post ‐ hoc, the different letters show significant among factor (*p* < 0.05).

The C content in RO leaves was higher than that in EO, with the exception of the +N‐P treatment. Therefore, a significant interaction of SP*NI was observed (*p* < 0.05). Drought reduced the C content in RO, but it did not affect the C of EO (Figure 4), thus, WM affected differently the two species (SP*WM, *p* < 0.05). The WM and NI changed the C content over time: at the beginning of the experiment, the highest content was highlighted in both species ‐N + P, regardless of WM.

However, the C content in ‐N + P was influenced by WM on DOY 193, as it decreased more significantly in D (from 464.1 ± 9.8 to 430.5 ± 11.0 g kg^−1^ in EO, from 481.6 ± 7.0 to 452.9 ± 12 g kg^−1^ in RO) than in WW (from 460.9 ± 8.0 to 447.03 ± 17.7 g kg^−1^ in EO, from 485.2 ± 4.3 to 464.5 ± 8.0 g kg^−1^ in RO), between DOY 174 and 193. Conversely, on DOY 213, WW ‐N + P reached values similar to those shown by D ‐N + P. As a matter of fact, a considerable effect of WM*NI*T interaction was observed.

In all stress stages, the N content was higher in EO leaves (30.5 ± 3.1 g kg^−1^) compared to RO (24.9 ± 3.1 g kg^−1^), regardless of NI (Figure S6). The N concentration in RO did not show differences over time, while a lower N content in EO was identified on DOY 193 (29.7 ± 3.4 g kg^−1^). The results indicated a decreasing trend of N in ‐N + P, in spite of species and WM.

**FIGURE 5:**
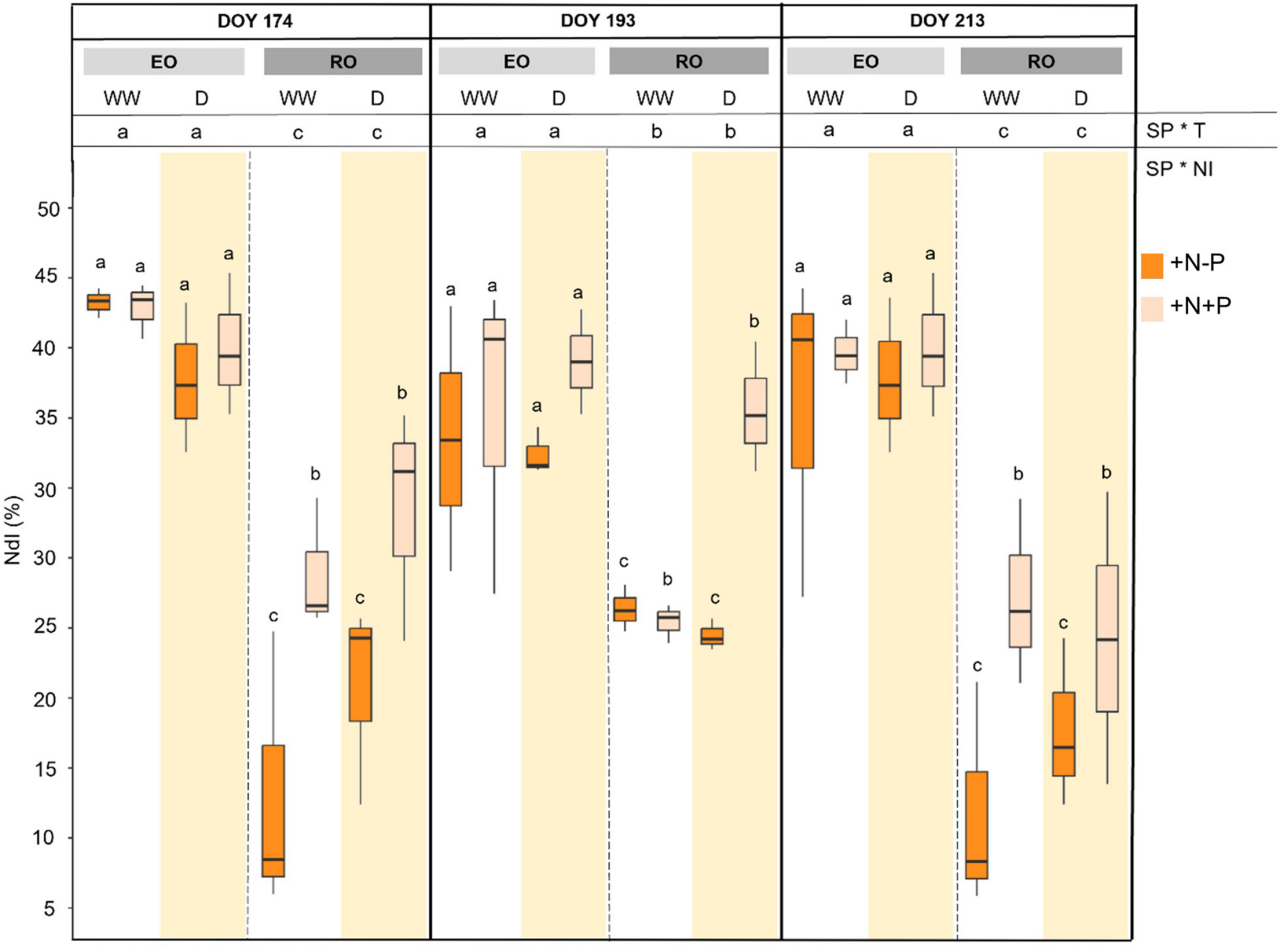
Nitrogen derived from Input (NdI) in leaves of both species (SP; English Oak, EO and Red Oak, RO) under different water management (WM; well‐watered, WW and drought conditions, D) with different nutrient input (NI), during time (T). According with ANOVA analysis and Tukey's post ‐ hoc, the different letters show significant among factor (*p* < 0.05).

The uptake of added N (NdI) was different between species, regardless of WM and NI: the lack of significance of NI is due to the fact that NdI refers to labelled nitrogen, thus only to +N‐P and + N + P treatments (Figure 5). The EO NdI uptake (42.3 ± 6.5%) was higher than that of RO (23.2 ± 9.4%). The NdI of the native species was not affected by NI, suggesting that P did not influence the N uptake. Conversely, the NdI of RO + N + P (28.1 ± 8.6%) was higher than +N‐P (18.3 ± 7.6%).

**FIGURE 6:**
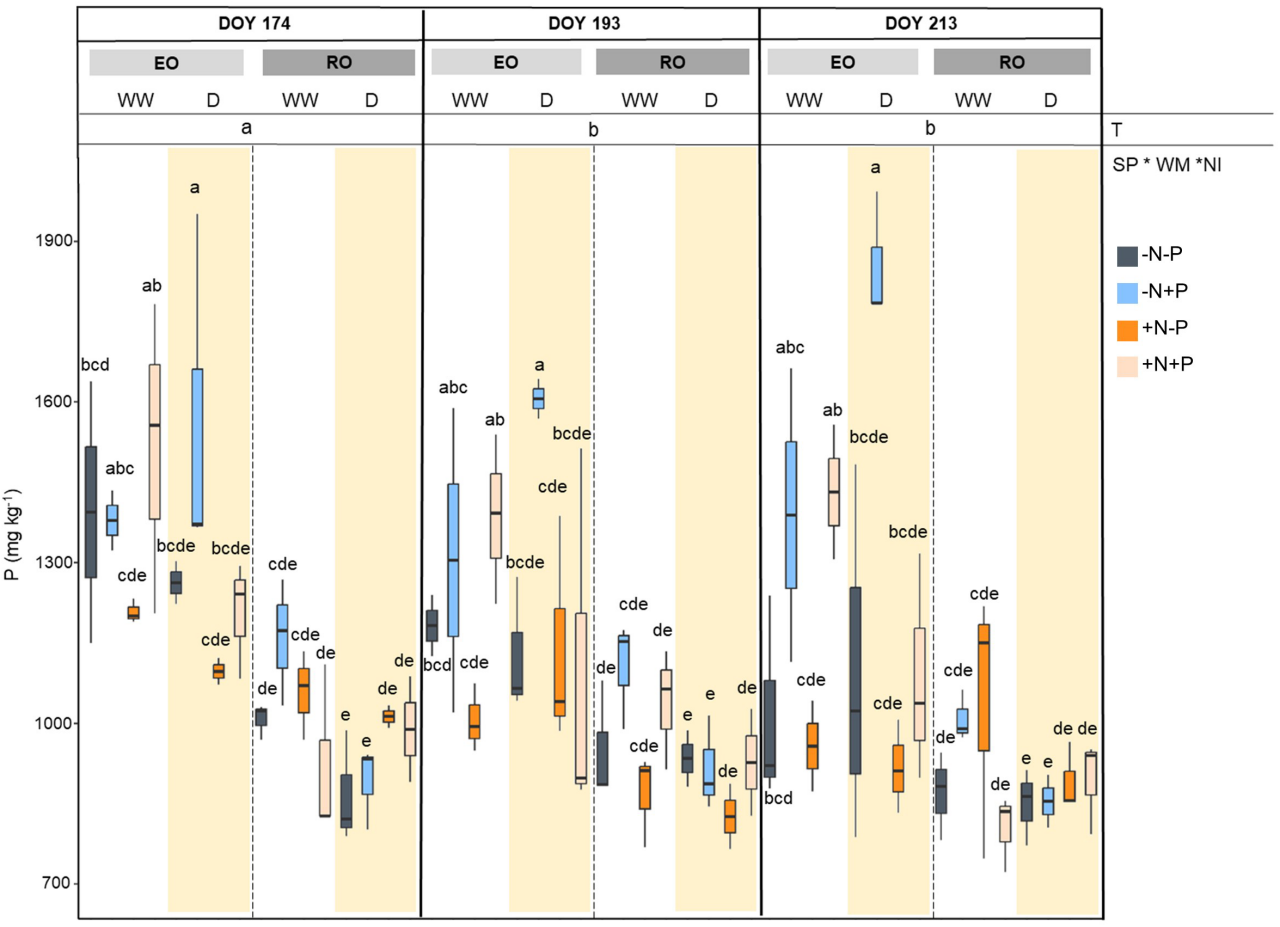
Phosphorous content (P) in leaves of both species (SP; English Oak, EO and Red Oak, RO) under different water management (WM; well‐watered, WW and drought conditions, D) with different nutrient input (NI), during time (T). According with ANOVA analysis and Tukey's post ‐ hoc, the different letters show significant among factor (p < 0.05).

The P content decreased over time regardless of SP, WM and NI (Figure 6). However, the P content in EO WW + N + P (1443.8 ± 185.0 mg kg^−1^) was higher compared to RO WW + N + P (921.0 ± 146.1 mg kg^−1^), as well as in D between EO ‐N + P (1674.2 ± 225.2 mg kg^−1^) and RO ‐N + P (887.2 ± 69.5 mg kg^−1^). As a matter of fact, ANOVA revealed the significance of time and SP*WM*NI. In EO D, the addition of combined N and P (+N + P) did not reveal an increased amount of P content, whereas higher P values were detected following the addition of P only (1674.2 ± 225.2 mg kg^−1^).

Calcium content in EO WW was 5511.7 ± 1246.2 mg kg^−1^, while the content in EO D decreased up to 4766.1 ± 1273.5 mg kg^−1^, as well as in WW (4614.4 ± 715.0 mg kg^−1^) and D RO (4678.2 ± 584.0 mg kg^−1^). Therefore, univariate ANOVA displayed significant differences in SP*WM (Figure S7). In addition, Ca concentration was remarkably affected by NI (*p* = 0.032), with higher values in ‐N + P (5364.8 ± 1262.1 mg kg^−1^) than in +N‐P (4564.2 ± 777.5 mg kg^−1^). The first drought cycles (DOY 174) reduced the Mg content in EO from 2801.5 ± 613.0 mg kg^−1^ (in EO WW) to up to 2453.4 ± 595.5 mg kg^−1^ (in EO D) (Figure S8). The significant effect of SP*NI*T has reduced the Mg content in EO ‐N + P, on DOY 213. During the experiment, the maximum Fe concentration was detected on DOY 174 in EO WW (613.72 ± 294.3 mg kg^−1^). Furthermore, regardless of the stress cycle, the native species showed a significant decrease of Fe in EO D ‐N‐P (2.68 ± 6.9 mg kg^−1^) compared to EO WW ‐N‐P (534.8 ± 537.30 mg kg^−1^), highlighting significant interaction of SP*WM*T and SP*WM*NI (Figure S9). Potassium content in plant tissues was lower in the water‐stressed alien species compared to the native one (Figure S10). As a matter of fact, the K content of RO D leaves was 2192.5 ± 1281.8 mg kg^−1^, while EO WW was characterized by 4256.1 ± 2516.1 mg kg^−1^and EO D by 3627.7 ± 2289.4 mg kg^−1^. On DOY 193, a decrease in K was identified in RO, conversely, EO showed the highest values. During water stress, both species exhibited an increase in K with N input alone or in combination with P. Although the Mn content in EO was around 2768.1 ± 889.7 mg kg^−1^, regardless of WM, RO D (2098.6 ± 875.9 mg kg^−1^) showed higher values than RO WW (1694.6 ± 705.1 mg kg^−1^). As a matter of fact, SP*WM significantly impacted the Mn content in leaves, showing notably reduced levels in RO (Figure S11). Additionally, the univariate ANOVA displayed the significance of time on the Mn content, indicating an increase from DOY 193. MANOVA analysis was performed for each species to understand the effect of WM and NI on the chemical composition of leaves. The results showed an effect of WM and NI on the variables for both species, but the significance of the WM*NI interaction was observed only on DOY 193 (Table S1).

### 3.3 Correlation between physiological parameters and chemical composition of leaves

**FIGURE 7:**
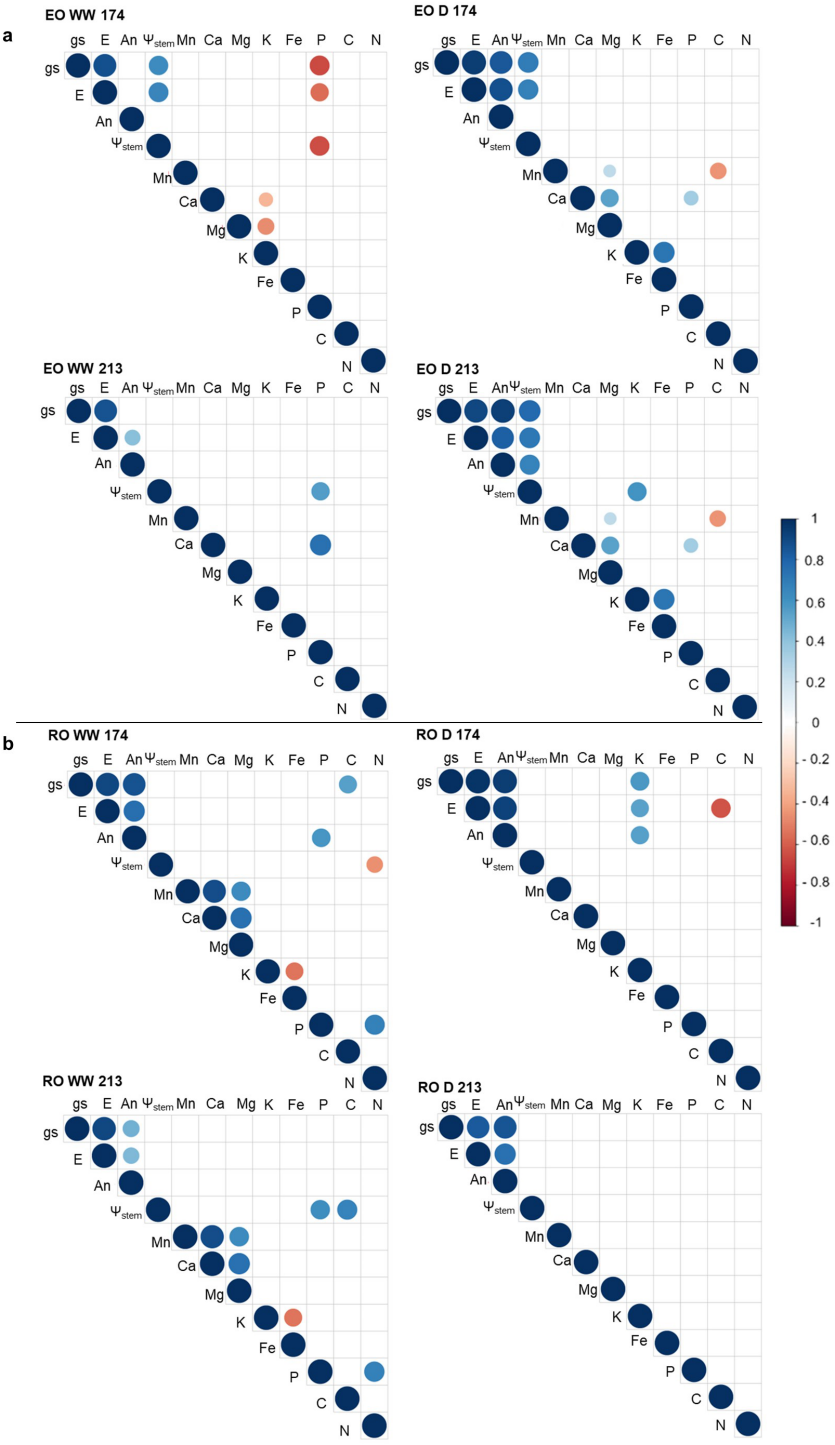
Spearman's correlation matrix between all the collected variables – physiological and chemical data – in EO (a) and RO (b), under well‐watered (WW) and drought conditions (D), during the first (DOY 174) and last stage (DOY 213). Only the correlation at a significance level *p*‐value ≤0.05 are reported.

**FIGURE 8:**
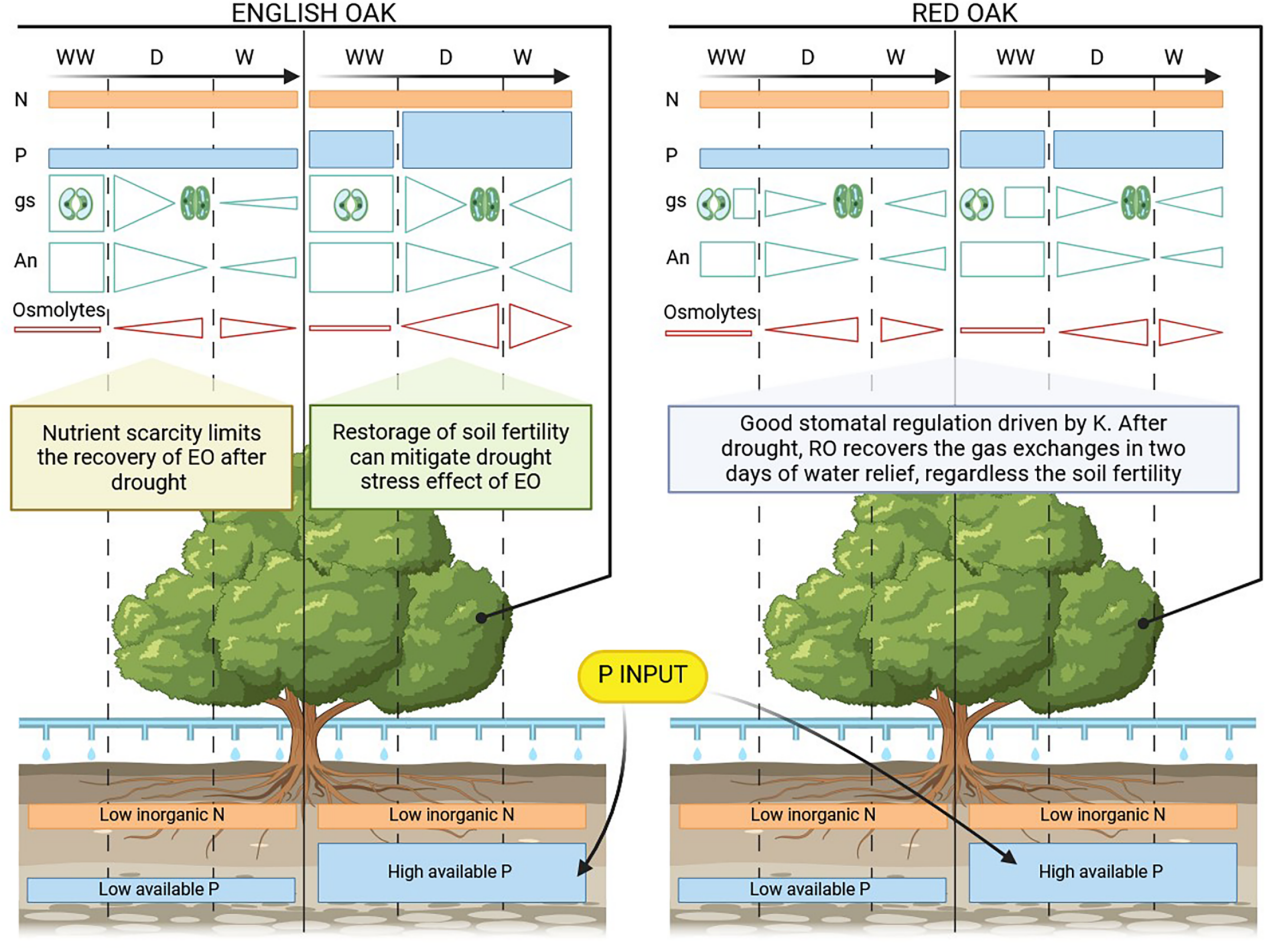
Graphical conclusions. The triangles highlight decreasing and increasing of parameters in English Oak (EO) and Red Oak (RO), under well‐watered (WW), drought (D) conditions and after two days of water relief (W).

The correlation matrix between physiological parameters and chemical composition of leaves was performed on each species, dividing plants between WW and D on the first (DOY 174) and last (DOY 213) stress treatments (Figure 7a & b, 8).

The correlogram of EO WW showed a strong positive correlation between gs and E in both stages. During the first stress, both parameters were positively correlated to Ψ_stem_, whereas a positive correlation was observed between A and E only in the last stage. On DOY 174, the P content in EO WW leaves was negatively correlated to gs, E and Ψ_stem_, while only a positive correlation between P and Ψ_stem_ was displayed on DOY 213. In the last stage, we observed that Ca was positively correlated to P, while on DOY 174, no correlation was discovered between them. As a matter of fact, the first drought stage showed that Ca, as well as Mg, were negatively correlated to K.

Physiological parameters of EO D were positively correlated on both stages, with the exception of An with Ψ_stem_ on DOY 174. Correlograms of both EO D 174 and EO D 213 displayed a significant positive correlation of P and Mg with Ca, as well as a positive relation between Mg and Mn. Conversely, a negative correlation between C and Mn was revealed only on DOY 174. Moreover, K of EO D was positively correlated with Fe and Ψ_stem_ in the first and last stages, respectively.

With RO, a remarkable positive correlation between gas exchanges was found, regardless of T and WM. On both DOY 174 and 213, P of RO WW was positively correlated to N, as well as Ca and Mn; Mg was related to Ca and Mn. Conversely, a negative correlation between K and Fe was detected. On DOY 174, RO WW displayed a negative correlation of N with Ψ_stem_ and a positive correlation between P and An, C and gs. Finally, on DOY 174, RO D displayed a positive correlation between K and gas exchanges but a negative correlation of C with E.

## 4 DISCUSSION

### 4.1 Physiological responses of well‐watered and drought‐stressed plants under nutrient‐deficiency conditions

Our results showed interspecific differences between RO and EO regarding leaf gas exchanges. Notably, under WW conditions, gs, E and An were higher in EO compared to RO, although the values of stem water potential were similar between the two species. Jacobs et al. (2009) observed similar leaf gas exchange values in RO seedlings under the same water management conditions, while EO values were consistent with those reported by Kebert et al. (2023). In our experiment, leaf gas exchanges and Ψ_stem_ of both species, as expected, declined as soil moisture content decreased, although EO D samples highlighted the greatest decline when compared to the corresponding WW condition. Interestingly, our findings agree with the results reported by Epron and Dreyer (1993), who previously explored the physiological effects of drought on EO saplings. However, a copious number of prior investigations on *Quercus* spp. elucidated a coordinated response of gs, An, E and Ψ_stem_ under water stress conditions and, in particular, the main role of gs in influencing all these parameters in case of limited water availability (Fotelli et al., 2000; Baquedano & Castillo, 2006; Gallé et al., 2007). Stomatal closure leads to the decline of E and CO_2_ assimilation (which negatively impacts the overall An), thus representing a drought‐avoidance strategy to enhance plant tolerance to water deficit (Damour et al., 2010; Roman et al., 2015; Urli et al., 2015). Although both species exhibited similar physiological responses when subjected to drought, RO recovered faster than EO in less fertile soils, confirming the hypothesis that RO plants, due to their lower water and nutrient demands, can recover more efficiently from drought events. Moreover, according to the literature (Hasenauer et al., 2017; Dyderski et al., 2018), our results suggest that RO was better adapted to dry conditions and that, in this experiment, the low nutrient availability of the soil did not limit the plant ability to face drought events.

### 4.2 The role of N and P and their interplay on plant drought responses

The NI did not avoid the water stress in either species, and this was confirmed by the Ψstem measurements. However, after the first drought event, NI promoted the recovery of EO only (EO W ‐N + P), while the physiological responses of RO were not affected by NI. It was already stated that the water and nutrient demand of RO is lower than EO (Miltner et al., 2016; Riepšas & Straigyte, 2008), suggesting a better adaptation of RO to nutrient and water availability changes, as confirmed by our results. However, the resilience of EO to water stress can be increased by the addition of P. Previous studies reported the beneficial effect of P to improve the antioxidant defence mechanism of plants, limiting the oxidative stress caused by water deficit conditions (Tariq et al., 2018; Wu et al., 2018). Therefore, the P input might help the recovery of EO likely because during dry events, the nutrient can limit the production of ROS in EO, thus restricting the protein degradation, lipid peroxidation, DNA fragmentation, and cell death (Apel & Hirt, 2004) The Spearman matrix highlighted significant correlations between gs and K^+^ in RO D. Taiz and Zeiger (2006) and Shabala and Pottosin (2010) described the crucial role of K^+^ during water stress, as a regulator of the osmotic potential and the opening/closing of stomata through potassium channels in stomatal guard cells. Therefore, the ability of RO to cope with drought may be related to a good ability to regulate stomatal aperture/closure and nutrient use efficiency.

Phosphorus supply could mitigate drought effects on EO. Under raised P availability conditions, plants can reduce gs, thus decreasing water loss (Goldstein et al. 2013). According to Mariotti et al. (2023), the high P content in EO plant tissues promotes the survival of this species under water stress. As a consequence, the P input in our low‐fertility soils may have reduced the negative effects of drought on EO due to the nutrient implications in photosynthesis processes (Singh et al., 2000; Jones et al., 2005) and osmolyte production (Tariq et al., 2019). A common strategy of drought‐stressed plants for cellular turgor maintenance is represented by the decrease of the osmotic potential through leaves osmolytes accumulation, thus changing the water potential gradient to retain water (and turgor) within the cells (Farooq et al., 2009). Tariq et al. (2018) observed that the addition of P on *Alnus cremastogyne* Burkill. under water stress‐induced increased levels of osmolytes, such as proline and soluble sugars. The P deficiency usually reduces photosynthesis due to the many cellular processes that depend on this element, including energy conservation, metabolic regulation, and signal production (Carstensen et al., 2018). Therefore, plants stressed by P scarcity increase the accumulation of reactive oxygen species (ROS), by‐products of photosynthesis whose excessive production can cause oxidative damage by reacting with biomolecules (e.g., peroxidation of membrane lipids), thus negatively impacting chloroplast functions (Weisz et al., 2017; Xia et al., 2021). For both EO and RO, ‐N + P treatment resulted in increased levels of C in leaves, probably due to an increase of photosynthates. In addition, P addition might have balanced the nutrient content in both plants, improving C content and limiting its starvation (Gessler et al., 2017). However, after the first drought stage, a general decrease in C content was observed until reaching – within each species ‐ similar values between D and WW plants. EO recovery was enhanced by the P supply but not when combined with N. The results from the +N + P treatment suggest that the addition of N alongside P could diminish the positive effect of P in treatments where both nutrients were added. The +N‐P input did not facilitate the recovery of either species and adversely affected the EO ability to restore the gas exchanges to pre‐stress levels. In fact, the parameters of EO W ‐N‐P and EO W + N‐P slowly increased after rewatering. Previous studies (Phillips et al., 2001; Scholz et al., 2007) have noted that N decreases the gs and E in conifers and broadleaves, and it increases the plant susceptibility to water deficit conditions (Mencuccini, 2003). Fusaro et al. (2016) observed a decrease in the ability of *Q. ilex* to cope with drought in response to N input. Therefore, our second hypothesis was partially confirmed because only P input, but not N or their combined addition, can mitigate the negative effects of drought on EO.

According to our third hypothesis, the uptake of EO is greater than that of RO, as the nutrient use efficiency differs among species. However, the use efficiency of P may differ from that of N within/between species. Red oak is considered to have lower water (Riepšas & Straigyte, 2008) and nutrient demand (Miltner et al., 2016) than European native oaks. The fraction of plant N derived from mineral inputs (NdI) confirmed the adaptation of RO to low fertility soils, as the NdI of the alien species was lower than that of EO, regardless of whether N was added with or without P. Nevertheless, the NdI of alien species was positively modulated by P addition, showing higher NdI values in RO + N + P compared to RO + N‐P, in both WW and D plants. The P supply may unbalance nutrient content in RO, stimulating the uptake of the more available N forms, i.e. those derived from the added N (Tilman, 1982). However, N supply did not affect the P content in RO WW and D, suggesting that in the alien species, P use efficiency is better than N use efficiency. Conversely, the NdI of EO did not depend on P supply, likely because the species is limited by both N and P availability.

According to Gonzales and Yanai (2019), ‐N + P and + N + P input induced P accumulation in EO WW leaves. However, EO D showed increasing P only with ‐N + P input. Villar‐Salvador et al. (2013) reported lower levels of P in oak seedlings as the soil available N exceeded the sufficient levels for the species (150–200 mg N plants^−1^). Therefore, the addition of N under drought conditions may have been excessive compared to the requirements of EO, which could explain the lack of P increase in EO D + N + P. Since roots were not sampled, we cannot exclude the possibility of P accumulation in the belowground biomass of EO D + N + P. In fact, previous results demonstrated the accumulation of nutrients in fine roots with high N levels in drought‐stressed *Alhagi sparsifolia* Shap. seedlings (Zhang et al., 2020). Furthermore, results from past experiments reported that, under water stress conditions, plants increase P in the roots but not in the leaves to enhance water uptake (Gargallo‐Garriga et al., 2015, 2018). This could suggest that the addition of +N + P can increase the EO water requirement, resulting in P accumulation in the roots of D plants, conversely to what was observed for ‐N + P.

### 4.3 Ecological implications of physiological responses of altered competitiveness

While both species exhibited comparable physiological adaptations to drought, RO displayed a faster recovery capacity than EO under low fertility conditions. This result, in line with the previous studies of Riepšas & Straigyte (2008) and Miltner et al. (2016), may suggest a competitive advantage of RO in nutrient‐poor environments subjected to recurring drought events. In competitive contexts, this difference may translate into greater resilience and colonization potential for RO in marginal and resource‐limited ecosystems. Conversely, the improvement in EO photosynthetic parameters under ‐N + P conditions indicates a higher benefit for the native species from P input, resulting in the mitigation of drought effects.

In a broader context, these observations supply valuable information for forest ecosystem management under drought or soil depletion conditions. In particular, the rapid recovery of RO after drought stress, combined with its ability to succeed under resource scarcity, could influence biodiversity dynamics, promoting the progressive diffusion of this alien species in ecosystems vulnerable to climate change or characterized by degraded soils, where EO is less competitive. On the other hand, although improving soil fertility could favour EO, careful management of N input is needed, as an excess of this nutrient may fail to provide the abovementioned advantages and could even increase the species susceptibility to environmental stress (Fusaro et al., 2016; Villar‐Salvador et al., 2013).

## 5 CONCLUSIONS

In this study, the response of two *Quercus* spp. (EO and RO) The combined effects of drought and nutrient inputs (N and P) were investigated. Physiological and chemical analyses of the leaves revealed distinct, species‐specific responses to repeated cycles of drought and rewatering. Read oak plants showed faster recovery in leaf gas exchanges, which was linked to effective stomatal regulation and a positive correlation between K and gs, regardless of N and P supply. This gives RO a competitive advantage in low‐resource environments, especially under repeated drought conditions. Conversely, the recovery of EO plants was slower, although P supply helped mitigate drought stress by enhancing photosynthesis and osmolyte production. This highlights that EO vulnerability can be particularly expressed in nutrient‐limited or drought‐prone environments under P‐deficient conditions. However, N input interfered with the recovery of EO, diminishing the positive effect of P and finally reducing its competitiveness relative to RO.

## AUTHOR CONTRIBUTIONS

All authors contributed to the study's conception and design. Conceptualization: Morena Rolando, Francesca Secchi, Luisella Celi. Data curation and visualization; Morena Rolando, Paola Ganugi, Luisella Celi, Francesca Secchi. Formal analysis and investigation: Morena Rolando, Paola Ganugi, Francesca Secchi, Luisella Celi. Methodology: Morena Rolando, Luisella Celi, Francesca Secchi.

## FUNDING ACQUISITION

European Union Next‐Generation EU (PIANO NAZIONALE DI RIPRESA E RESILIENZA (PNRR) – MISSIONE 4 COMPONENTE 2, INVESTIMENTO 1.4 – D.D. 1032 17/06/2022, CN00000022), Spoke 6. Supervision: Luisella Celi, Francesca Secchi. Project administration: Luisella Celi. Writing ‐ original draft: Morena Rolando, Paola Ganugi. Writing ‐ review & editing: all authors.

## EQUATIONS

Equation (1) Equation to calculate the nitrogen derived from mineral inputs.

Equation (2) Equation to calculate the delta recovery of each physiological parameter for each recovery stage and species.

Equation (3) Equations to calculate the recovery of each physiological parameter on a specific recovery stage of W.

Equation (4) Equations to calculate differences of each physiological parameter in WW samples during the recovery stage of W plants.

## Supporting information


**Figure S1:** Leaf transpiration (E) of both species (SP; English Oak, EO and Red Oak, RO) under different water management (WM; well‐watered, WW and drought conditions, D) with different nutrient input (NI), during time (T). According with ANOVA analysis and Tukey's post ‐ hoc, the different letters show significant among factor (*p < 0.05*).
**Figure S2** Leaf transpiration (E) of both species (SP; English oak, EO and Red oak, RO) under different water management (WM; well‐watered, WW and rewatered after drought, W) with different nutrient input (NI), during time (T). According with ANOVA analysis and Tukey's post ‐ hoc, the different letters show significant among factor (*p < 0.05*).
**Figure S3** Figure S3 Delta recovery rate of gs (∆gs) of both species (SP; English oak, EO and Red oak, RO) rewatered after drought (W), with different nutrient input (NI), during time (T). According with ANOVA analysis and Tukey's post ‐ hoc, the different letters show significant among factor (*p < 0.05*).
**Figure S4** Delta recovery rate of E (∆E) of both species (SP; English oak, EO and Red oak, RO) rewatered after drought (W), with different nutrient input (NI), during time (T). According with ANOVA analysis and Tukey's post ‐ hoc, the different letters show significant among factor (*p < 0.05*).
**Figure S5** Delta recovery rate of An (∆An) of both species (SP; English oak, EO and Red oak, RO) rewatered after drought (W), with different nutrient input (NI), during time (T). According with ANOVA analysis and Tukey's post ‐ hoc, the different letters show significant among factor (*p < 0.05*).
**Figure S6** Nitrogen content in leaves (N) of both species (SP; English oak, EO and Red oak, RO) under different water management (WM; well‐watered, WW and drought conditions, D) with different nutrient input (NI), during time (T). According with ANOVA analysis and Tukey's post ‐ hoc, the different letters show significant among factor (*p < 0.05*).
**Figure S7** Calcium content in leaves (Ca) of both species (SP; English oak, EO and Red oak, RO) under different water management (WM; well‐watered, WW and drought conditions, D) with different nutrient input (NI), during time (T). According with ANOVA analysis and Tukey's post ‐ hoc, the different letters show significant among factor (*p < 0.05*).
**Figure S8** Magnesium content in leaves (Mg) of both species (SP; English oak, EO and Red oak, RO) under different water management (WM; well‐watered, WW and drought conditions, D) with different nutrient input (NI), during time (T). According with ANOVA analysis and Tukey's post ‐ hoc, the different letters show significant among factor (*p < 0.05*).
**Figure S9** Iron content in leaves (Fe) of both species (SP; English oak, EO and Red oak, RO) under different water management (WM; well‐watered, WW and drought conditions, D) with different nutrient input (NI), during time (T). According with ANOVA analysis and Tukey's post ‐ hoc, the different letters show significant among factor (*p < 0.05*).
**Figure S10** Potassium content in leaves (K) of both species (SP; English oak, EO and Red oak, RO) under different water management (WM; well‐watered, WW and drought conditions, D) with different nutrient input (NI), during time (T). According with ANOVA analysis and Tukey's post ‐ hoc, the different letters show significant among factor (*p < 0.05*).
**Figure S11** Manganese content in leaves (Mn) of both species (SP; English oak, EO and Red oak, RO) under different water management (WM; well‐watered, WW and drought conditions, D) with different nutrient input (NI), during time (T). According with ANOVA analysis and Tukey's post ‐ hoc, the different letters show significant among factor (*p < 0.05*).
**Table S1**. Multivariate analysis (MANOVA) based on Pillai's Trace test of water management (WM) and nutrient input (NI) on chemical characterization of leaves (C, Ca, Fe, K, Mg, Mn, N, P) of English oak (EO) and Red oak (RO) species under different stress time (DOYs 174, 193, 213).
